# From the Deep: Overlap of Neutrophilic Dermatoses Disorders Associated With Acute Myeloid Leukemia

**DOI:** 10.7759/cureus.33456

**Published:** 2023-01-06

**Authors:** Miguel Martins, Joana Barbosa, Sofia P Eusébio, Rita Prayce, Pedro Pires

**Affiliations:** 1 Internal Medicine, Centro Hospitalar Universitário de Lisboa Central, Lisbon, PRT; 2 Dermatology, Centro Hospitalar Universitário de Lisboa Central, Lisbon, PRT

**Keywords:** pathergy, hematological malignancies, autoimmunity, flt3-inhibitor, mediastinitis, neutrophilic panniculitis, acute myeloid leukaemia, pyoderma gangenosum, neutrophilic dermatosis

## Abstract

We report the case of a man in his 50s with refractory acute myelomonocytic leukaemia (AML) who presented with neck swelling, fever, and elevated levels of C-reactive protein (CPR) after venous punctures. An infected haematoma was presumed, but the patient showed no signs of improvement under broad-range antibiotics, and microbiological results were negative. The subsequent development of a rapidly evolving erythematous-violaceous plaque around a site that had previously punctured on the extensor surface of the right arm prompted us to reconsider the clinical setting as a whole and consider the hypothesis of deep neutrophilic dermatosis (ND) associated with haematologic malignancy. A biopsy of the arm lesion showed an aseptic neutrophilic infiltrate, confirming this diagnosis. The patient was initially treated with high-dose intravenous corticosteroids, resulting in a dramatic improvement of the skin lesions.

## Introduction

Managing febrile haemato-oncological patients, in whom inflammation plays a significant role in many aspects of disease progression [1], is a challenge that necessitates consideration of other diagnoses besides infection. This clinical case of neutrophilic dermatosis (ND) in a patient previously diagnosed with a rare form of acute myeloid leukaemia (AML) is a great example of that complexity and of how important it is to be vigilant of the new clinical findings in these patients with a dysfunctional immune system. Furthermore, to our knowledge, we report the first possible overlap of two different neutrophilic dermatoses, neutrophilic panniculitis (NP) and pyoderma gangrenosum (PG).

## Case presentation

This case is about a man in his 50s with known severe pancytopenia due to refractory acute myelomonocytic leukaemia (AMML), a rare form of AML, with FMS-like tyrosine kinase 3 internal tandem duplication (FLT3-ITD) and nucleophosmin (NPM1) mutations. The AMML was diagnosed in July 2019. It was initiated with induction therapy with cytarabine and idarubicin at the time, followed by consolidation therapy with cytarabine and mitoxantrone and CNS prophylaxis. The patient initially achieved remission, but three relapses ensued in the following two years: the first one in November 2020 (fludarabine, cytarabine, idarubicin, and granulocyte colony-stimulating factor were used for induction and consolidation therapy); the second relapse in April 2021 (treated with a time-sequential therapy regimen consisting of etoposide, mitoxantrone, and cytarabine, followed by consolidation therapy with venetoclax); the third relapse was in August 2021. Since then, the patient has been on the sixth cycle of gilteritinib with a favourable response (a myelogram and bone biopsy from February 2022 did not show the progression of the disease).

In March 2022, the patient was admitted to an internal medicine ward due to an initial episode of nonsevere Clostridioides difficile infection (CDI). One month before, the patient was hospitalised in a foreign country (during a trip) due to aggravated pancytopenia, severe neutropenia, and a presumably infected spontaneous haematoma; the patient´s report did not fully clarify the microbiological study. The patient received antibiotics for 21 days (days with endovenous piperacillin-tazobactam, followed by oral amoxicillin-clavulanic acid for seven days), with clinical resolution of the infection. The patient had been under treatment with gilteritinib (120 mg a day), a potent FLT3 inhibitor, for five months at the time, but decided to autonomously suspend it for two weeks during that previous hospitalization.

**Figure 1:**
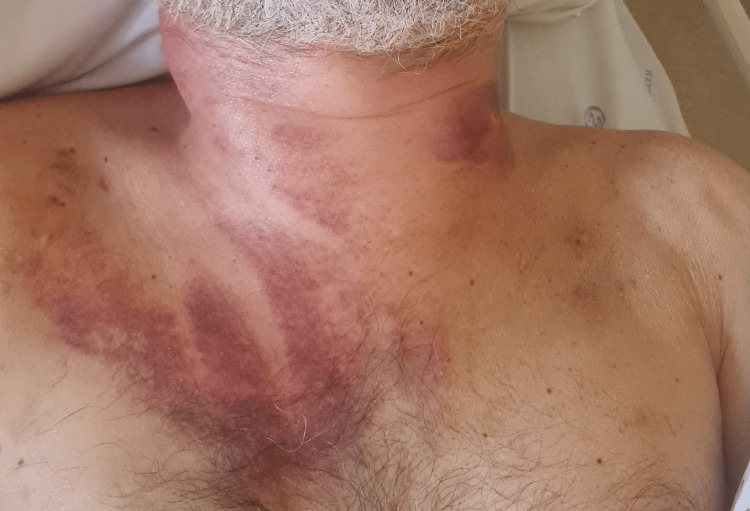
Local swelling and erythema in the neck region six days after venous punctures.

Four days after being admitted, due to the lack of peripheral venous access, recurring phlebitis, and worsening condition with progression to severe CDI, an internal jugular central venous catheter (CVC) insertion was attempted on both veins (right and left) but not accomplished as an iatrogenic complication, a discrete hemorrhagic suffusion. Six days later, the patient still had a fever, the inflammatory parameters were rising, and he still needed frequent red blood cells and platelet transfusions, even though the abdominal pain and diarrhoea had regressed. During that period, the patient’s neck developed local inflammatory signs, with growing localised swelling, tenderness, and redness around the previous CVC puncture sites (Figure 1).

Investigations

**Figure 2:**
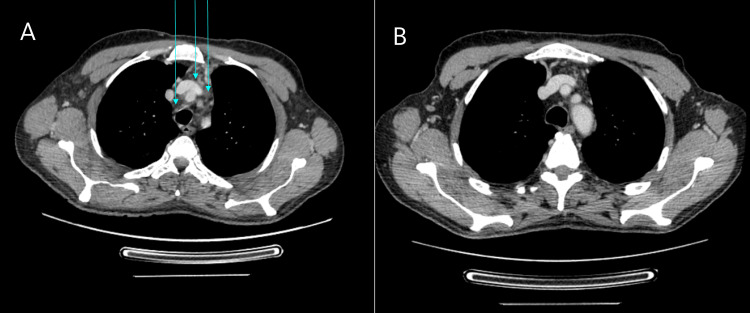
Axial view of the CT scan of the thorax (A) Six days after CVC puncture, this image shows densification of the structural adipose cell environment with multifocal impairment of the normal anatomical cleavage between structures (blue arrow) in the superior mediastinum. (B) The same location after 15 days of treatment with corticosteroids, with almost no changes.

**Figure 3:**
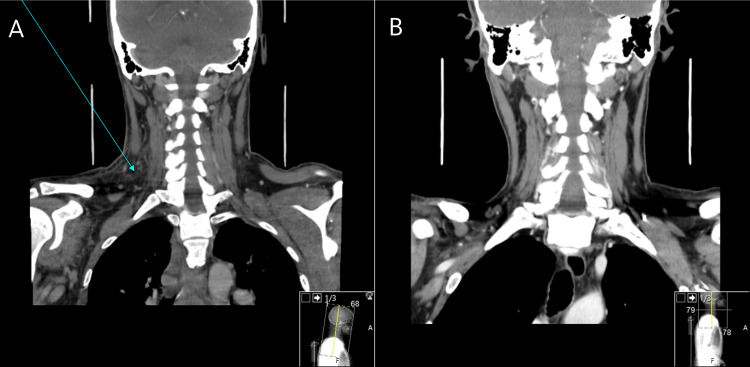
Coronal view of the CT scan of the neck (A) Six days after the CVC puncture, this image shows densification of the structural adipose cell environment with multifocal impairment of the normal anatomical cleavage between structures (blue arrow) below the sternocleidomastoid muscle. (B) The same location after 15 days of treatment with corticosteroids, with almost no changes.

A contrast-enhanced neck CT scan was performed, demonstrating important bilateral and asymmetric thickening of the sternocleidomastoid muscles, which were frankly heterogeneous with spontaneously hyperdense aspects in their interior, suggesting intra-muscular haemorrhage with clear densification of the visceral space adipose tissue and concomitant hyperdensities of fat and fascial spaces due to interstitial oedema. It also revealed densification of the superior mediastinal adipose planes, compatible with mediastinitis (Figure 2A and Figure 3A).

**Figure 4:**
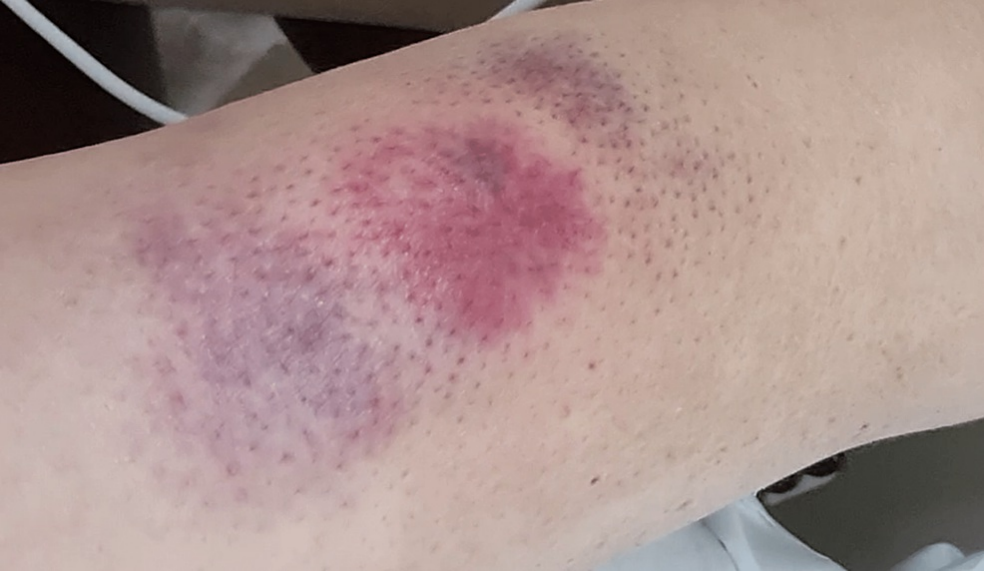
Local swelling and erythema a few hours after a venous puncture in the patient’s right arm.

Laboratory examination disclosed elevated levels of C-reactive protein (CRP) of 197,4 mg/L (< 5.0 mg/L), an elevated erythrocyte sedimentation rate of 122 mm/hr (0-22 mm/hr), minimally elevated levels of procalcitonin of 0,35 ng/mL (suggestive of infection > 0,25 ng/mL), and aggravating pancytopenia (haemoglobin lowered from 7,8 to 6,9 g/dl and platelets from 41 x 109/L to 16 x 109/L in two days), except for leukocytes (4,07 x 109/L), which were under the normal value (4,50 x 109/L), but almost double compared to values from the previous day (2,17 x 109/L), mainly due to an increase in neutrophils ( from 1,30 x 109/L to 2,76 x 109/L). Aspartate aminotransferase, alanine aminotransferase, lactate dehydrogenase, creatine kinase, and myoglobin levels were within normal ranges. Serum protein electrophoresis and immunoelectrophoresis did not reveal monoclonality. An infected haematoma was presumed. After seven days of empirical treatment with meropenem and linezolid, no microbiological agent was identified in blood cultures, and the patient maintained a swollen neck with local inflammatory signs. Laboratory examination still revealed high CRP levels (144,2 mg/L) and procalcitonin (PCT) levels (0,5 ng/mL), with persistent pancytopenia. Keeping in mind the patient's immunosuppression and relevant pancytopenia, it was decided not to proceed with further invasive studies such as aspiration or biopsy at that moment. On that same day, the patient complained about pain in his right upper arm, with local swelling and erythema around a site that had previously been punctured on the extensor surface of the right arm (Figure 4).

**Figure 5:**
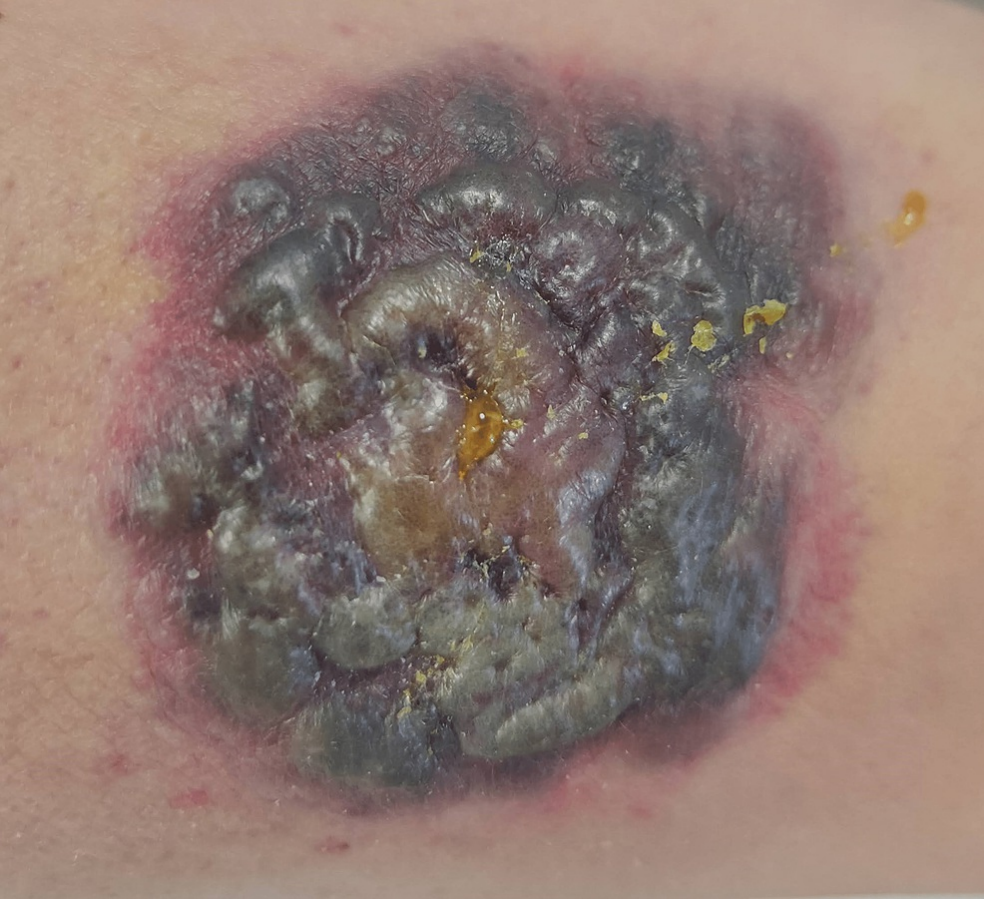
An erythematous-violaceous plaque of circular configuration with well-defined borders, an irregular, cerebriform surface, and a surrounding erythematous halo.

On the next day, an erythematous-violaceous plaque with a circular configuration, well-defined borders, an irregular surface, and a cerebriform shape with a surrounding erythematous halo measuring 7 cm in diameter developed at that location (Figure 5).

**Figure 6:**
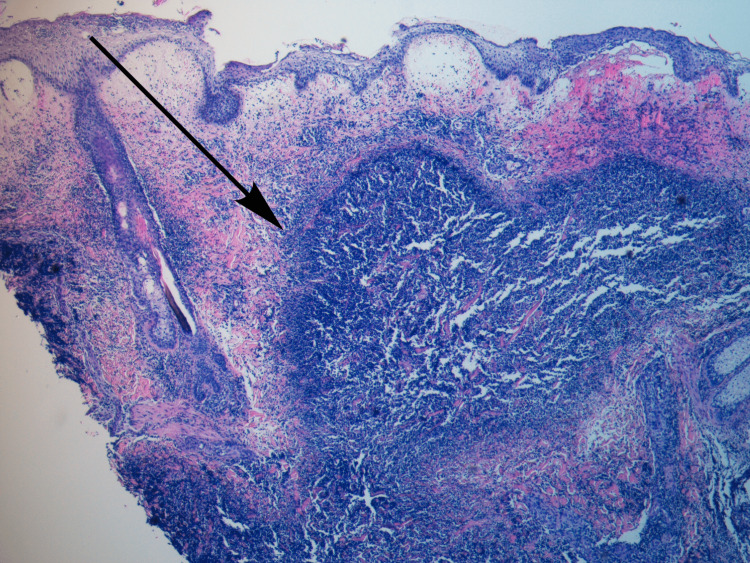
Histological slide from affected skin revealing a dense neutrophilic infiltrate in the reticular dermis extending to the hypodermis (arrow).

The patient was evaluated by dermatology, and a punch skin biopsy of the arm lesion was performed. The histopathological study of the biopsy, after performing hematoxylin and eosin staining, showed confluent epidermal necrosis and keratinocyte pallor and oedema in the lower half of the spinous layer and basal layer; prominent oedema in the superficial dermis, with subepidermal dislocation; and a dense infiltrate of neutrophils with perivascular accentuation and leukocytoclasis, without evidence of vasculitis, extending to the hypodermis (Figure 6).

Microbiological cultures for bacteria, mycobacteria, and fungi from lesional skin yielded negative results.

Differential diagnosis

Due to the patient’s immunosuppressive state with severe neutropenia, a bacterial infection of haematomas or infective panniculitis was presumed as the cause of neck swelling. Infective cellulitis could explain the skin lesion. Fungal or mycobacterial infections were deemed improbable due to the acute installation of the symptoms after iatrogenic punctures. Nevertheless, no microbiological agent was found either in blood or tissue cultures, and the patient never improved under broad-spectrum antibiotics. An infectious process was excluded as a possibility to explain the whole clinical picture.

Leukaemia cutis, or infiltration of the skin with neoplastic leukocytes, can be observed during myelogenous leukaemia and is correlated with a poor prognosis. It displays in a variety of ways, including bullae, although the classical presentation is violaceous to red-brown papules, plaques, or nodules. Histologically, skin infiltrates consist of malignant immature myeloblasts without neutrophils, which were not found in the biopsy, and therefore this diagnosis was excluded.

The abrupt onset of the cutaneous lesion and the presence of systemic inflammatory signs with no evidence of infection, as well as an underlying haematological malignancy, suggested the hypothesis of a neutrophilic dermatosis (ND). The dermal neutrophilic infiltrate favoured that diagnosis. FLT-3 inhibitors, such as gilterinib, which was reinitiated by the patient two weeks prior to this admission, have been associated with NDs [2-5]. We can hypothesise the contribution of this drug to the whole clinical picture, supporting the diagnosis of ND 2-4. The cutaneous lesion morphology, with peripheral erythema, an undermining border, and tenderness, along with rapid progression and a history suggestive of pathergy indicated the diagnosis of pyoderma gangrenosum (PG), a deep ND. Neutrophilic dermatoses can also be a manifestation of Behçet syndrome (BS), and pathergy is a relatively common clinical feature that can contribute to the diagnosis of this syndrome. BS does not usually present as a paraneoplastic syndrome, although patients with this syndrome appear to have a higher risk of being afflicted with malignancy, namely haematological and thyroid cancer. However, the described patient did not have a history of previous recurrent oral or genital ulcers or any ocular, neurologic, vascular, or other systemic manifestations that would make us consider a previously missed diagnosis of BS. Further laboratory investigations with serum autoantibodies or colonoscopy were not pursued due to the already-known diagnosis of a systemic disease associated with PG and the lack of other clinical findings suggestive of an autoimmune disease.

Possible neutrophilic panniculitis (NP), another condition categorised as deep ND, was assumed to be the cause of the inflammatory process surrounding the neck and superior mediastinum region for the following reasons: the almost concurrent onset of both NP and PG, the continued ineffectiveness of broad-spectrum antibiotic therapy, and the findings of fat tissue hyperdensity on the CT scan, suggestive of an inflammatory process. Due to the lack of a skin biopsy, we could not confirm this diagnosis. Neutrophilic myositis is also described in cases of PG, but the patient didn’t have muscular weakness, and laboratory markers of muscle lesions were normal.

Treatment

The patient initiated systemic corticosteroid (CS) therapy with intravenous methylprednisolone 1 mg/kg/day. Wound care with collagenase and hyper-absorbent dressings was also implemented. The initial response was excellent, both clinically (with apyrexia and subduing pain, skin lesions, and neck swelling) and based on lab results (with a drastic reduction of CRP to 45.1 mg/L just two days after initiation). After three days, the CS was changed to oral prednisolone 1 mg/kg/day.

The patient was discharged from our unit 15 days after the initiation of CS with sustained apyrexia, no oedema or resolving skin lesions, and normal CPR and PCT levels. The CT scan revealed clear improvement, with virtually no signs of haematoma or oedema (Figure 2B and 3B).

Outcome and follow-up

The patient continued his follow-up in the clinical haematology outpatient clinic. The prednisolone was gradually tapered off over eight weeks with complete resolution. He had no recurrences of pyoderma gangrenosum during the ensuing five months. Gilteritinib treatment was later stopped because of disease progression, and the patient initiated venetoclax and azacitidine. Five months later, after being dismissed from our unit while waiting for a therapeutic response to proceed to a bone marrow transplant, the patient was admitted to another hospital and died of severe abdominal septic shock. In the meantime, no new skin lesions appeared.

## Discussion

This case illustrates the complexity of managing patients with haematological malignancies (HMs), in which the immunological imbalance leads to both pro-inflammatory and immunosuppressive states. A prospective multicentre study in France reported that 77% of febrile episodes in patients with AML represented documented infections, leaving around one-quarter of all cases of fever of unknown origin due to several other causes, secondary to drugs, the transfusion of blood products, the underlying disease, or inflammatory disorders [6]. Several cutaneous immune-mediated disorders have been shown to occur in patients with HMs, such as autoantibody-mediated skin diseases, vasculitis, granulomatous dermatoses, eosinophilic dermatoses, and, as illustrated in this clinical case, neutrophilic dermatoses (NDs). Myeloid malignancies, such as AML, are the HMs most frequently associated with NDs [7], and their onset may follow, precede, or be concomitant with the ND's diagnosis.

NDs are a heterogeneous group of inflammatory conditions characterised by polymorphic cutaneous lesions resulting from a neutrophil-rich tissue infiltrate in the absence of infection. In these disorders, the possible involvement of almost any organ system has led to the coining of the term "neutrophilic diseases" [8]. NDs are frequently associated with a variety of systemic disorders besides HMs, such as inflammatory bowel disease (IBD) and rheumatoid arthritis (RA). The pathogenesis of these disorders is still being clarified. NDs share the same pro-inflammatory effectors also found in autoinflammatory syndromes (for example, familial Mediterranean fever), suggesting common physio-pathological mechanisms [9]. An overexpression of proinflammatory cytokines (Interleukin (IL)-1, IL-8, IL-17, and tumour necrosis factor-α) leading to increased neutrophil recruitment and accumulation in the skin is considered the primary step in the pathophysiological scenario [10]. Concerning myeloid neoplasm-associated NDs, there’s mounting evidence that skin-infiltrating neutrophils may be clonally related to the neoplastic cells and may have differentiated from them [11], with some authors even suggesting that leukaemia cutis and Sweet syndrome (SS) associated with AML may be included in the same disease spectrum. Moreover, NDs, especially SS but also PG and neutrophilic eccrine hidradenitis, may be induced by drugs used to treat AML, in particular FLT3 inhibitors such as gilteritinib [2-5].

NDs encapsulate a variety of conditions and can be segmented into three core groups based on their clinicopathological distinctions: I. deep or hypodermal forms, exemplified by pyoderma gangrenosum (PG) and neutrophilic panniculitis (NP); II. plaque-type or dermal forms, epitomised by Sweet syndrome (SS); III. superficial or epidermal forms, such as subcorneal pustular dermatosis [8]. Overlap forms between the different entities have also been described in association with underlying HMs [8].

Our case report presents a patient with AML who recently restarted an FLT3 inhibitor, gilteritinib, with two different conditions of deep ND: a possible NP affecting the neck and superior mediastinum regions and a PG in his right arm.

NP is characterised by painful, inflammatory subcutaneous masses and clinically manifests as deep-seated, erythematous, tender nodules or plaques, classically involving the arms, legs, and trunk. It is not a well-known condition, although it has been reported to be associated with rheumatoid arthritis (RA) [12], Crohn's disease [13], myelodysplastic syndromes (MDS) [14], and chronic myelogenous leukaemia (CML) [15]. A skin biopsy is essential for the diagnosis and should be obtained by a deep incisional biopsy to obtain an adequate sampling of subcutaneous fat. Histology reveals a neutrophilic infiltrate in the subcutaneous fat, predominating in the lobules with mild necrosis. Besides infection, neutrophilic panniculitis must be distinguished from pancreatic panniculitis, 1-antitrypsin deficiency-associated panniculitis, erythema induratum, and early erythema nodosum. Oral steroids seem to be highly effective in NP, although dapsone, an IL-1 antagonist, or a TNF antagonist may be needed as a steroid-sparing drug or in refractory cases.

PG is a rare, debilitating inflammatory skin disease with variable clinical manifestations and is therefore divided into various subtypes: classic, vegetative, pustular, bullous, peristomal, and postoperative. The incidence of PG is estimated to be six patients per million people per year [16], occurring at any age but most commonly between 40 and 50 years, with a possible slight female predominance, although it’s possible men are more commonly affected in malignancy-associated PG and have a worse prognosis [17]. Around 33% of PG cases are associated with other immune-mediated diseases, most commonly IBD (20.2%), RA or inflammatory arthritis (11.8%), and HMs (8.9%) [16].

Classic or ulcerative PG is characterised by a painful, rapidly evolving central ulcer with an undermined, irregular, erythematous-violaceous edge. A primary lesion begins as a deep-seated, painful nodule or as a superficial hemorrhagic pustule, often following minor trauma (for example, venous puncture), a phenomenon known as pathergy that is present in around one-third of the cases of PG [18]. This lesion quickly undergoes necrosis, leading to a central ulceration that discharges a purulent exudate; the irregular border is elevated and is dusky-red or purplish; it is soggy and often perforated so that pressure releases pus, both into the ulcer and through the openings. Habitually, during treatment, vegetative lesions may develop on the surface of a previously classic PG, while pustular lesions normally precede or are concomitant with PG ulcers. Atypical or bullous PG is identified by blisters that are present at onset and later evolve into ulcerative lesions.

A definitive diagnosis of PG can be challenging, so maintaining a high index of suspicion for the disease is crucial to making the diagnosis. There have been three suggested diagnostic frameworks to help aid the diagnosis of classic PG: the Su criteria, the Delphi consensus criteria for ulcerative PG, and the PARACELSUS score [19-21] (Table 1).

**Table 1:** Assessment of three diagnostic criteria for pyoderma gangrenosum PG: pyoderma gangrenosum; IBD: inflammatory bowel disease; RA: rheumatoid arthritis; VAS: visual analogue scale for pain

	Su criteria (2004) [19]	Delphi consensus criteria for ulcerative PG (2018) [20]	PARACELSUS score (2018) [21]
Major criteria	Rapid progression of a painful, necrotic cutaneous ulcer with an irregular, violaceous, and undermined border. Other causes of cutaneous ulceration have been excluded.	Biopsy with neutrophilic infiltrate	The progressive course of the disease. The absence of relevant differential diagnoses. Reddish-violaceous wound border (each three points)
Minor criteria	History is suggestive of pathergy or a clinical finding of cribriform scarring. Systemic disease is associated with PG (IBD, RA). Histopathologic findings of sterile dermal neutrophilia ± mixed inflammation ± lymphocytic vasculitis. Treatment response (generally a rapid response to systemic therapy)	Exclusion of infection on histology; pathergy; a personal history of IBD or inflammatory arthritis; a papule, pustule, or vesicle that rapidly ulcerates; peripheral erythema, an undermining border, and tenderness at the site of ulceration; cribriform or "wrinkled-paper" scars at healed ulcer sites; decrease in ulcer size after immunosuppressive treatment	Amelioration due to immunosuppressants; characteristically bizarre ulcer shape; extreme pain > 4 (VAS); localised pathergy phenomenon (each two points). Additional criteria: suppurative inflammation in histopathology, undermined wound margin, associated systemic disease (each one point)
Diagnosis	Both major and ≥ two minor criteria	Major criteria and ≥ four minor criteria	Highly likely PG >10 points

A recent comparative study seems to show that the PARACELSUS score identified the highest proportion of patients in a PG cohort, but further research is needed [22]. Their main discrepancies reside in the approach to ruling out alternative diagnoses and the importance of requiring a neutrophilic infiltrate on histopathology. The lack of validated criteria, combined with the absence of specific laboratory or histopathological indicators, leaves patients with PG at risk of delayed diagnosis and mismanagement. PG should be considered in patients whose cutaneous lesions are painful, rapidly expanding, unresponsive to antibiotics, or worsening with surgical debridement, especially if pathergy is present. Vigilant monitoring for PG is also appropriate in any patient with a frequently associated systemic disease presenting with new skin lesions.

The treatment of pyoderma gangrenosum is often challenging. Systemic CS (dose 0.5-1 mg/kg/day) has a quick onset of action, making it the first-line treatment. Only 40% of patients achieve complete remission. In severe cases, it is recommended to combine systemic CS with immunosuppressive adjuvants, with cyclosporine being indicated as the first-line agent [23]. In mild cases (ulcer lesion < 3cm), topical therapy with CS or calcineurin inhibitors may suffice. In refractory cases, new effective targeted therapies with biologics (preferably anti-TNF-α agents) are advised. Besides medical therapy, wound care and pain control are crucial in the management of PG cases.

And if the clinical distinction between a skin infection and neutrophilic dermatosis wasn’t enough of a challenge, the initial clinical picture, in this case, was hidden deep underneath. Besides the clinical and histopathological findings being consistent with PG, the patient described in our clinical case met the criteria for PG in all the previously suggested diagnostic frameworks. It is not possible to conclude that our patient had a definitive diagnosis of NP in the neck and superior mediastinum, though that possibility was inferred due to the clinical picture, the hyperdensity in the fat tissue seen in the CT scan, the absence of response to antibiotics, the posterior emergence of an ND, and the rapid remission after the initiation of CS. Pathergy was another clue that both manifestations were connected and showed the magnitude of the exaggerated inflammatory response in ND and how systemic it is. Internal medicine must be ready to diagnose and manage ND, as a great number of patients with ND suffer from other underlying systemic conditions.

## Conclusions

This clinical case is a clear representation that fever in patients with haematological malignancies does not always mean infection. Other causes must be investigated, such as autoimmune or autoinflammatory disease or drugs, especially in the absence of a response to antibiotics, and neutrophilic dermatosis is one of the potential sources of fever. Neutrophilic dermatoses are usually associated with a systemic disease. Therefore, internists, rheumatologists, and haematologists should be prepared to recognise and treat these conditions. Neutrophilic dermatoses act as systemic autoinflammatory diseases, and overlapping conditions are more common in patients with haematological malignancies. Pathergy phenomena should alert us to a possible neutrophilic dermatosis, such as Sweet syndrome, Behçet syndrome, or, as demonstrated in this clinical case, pyoderma grangrenosum. It’s also important to recognise FLT-3 inhibitors, such as gilteritinib, as potential causative agents of neutrophilic dermatosis, although not enough evidence is available to determine gilteritinib as the definitive cause of neutrophilic dermatosis in this clinical report.

## References

[1] Pietras EM (2017). Inflammation: a key regulator of hematopoietic stem cell fate in health and disease. Blood.

[2] (2022). Xospata, INN-gilteritinib. https://www.ema.europa.eu/en/documents/product-information/xospata-epar-product-information_en.pdf.

[3] Fathi AT, Le L, Hasserjian RP, Sadrzadeh H, Levis M, Chen YB (2013). FLT3 inhibitor-induced neutrophilic dermatosis. Blood.

[4] Varadarajan N, Boni A, Elder DE, Bagg A, Micheletti R, Perl AE, Rosenbach M (2016). FLT3 inhibitor-associated neutrophilic dermatoses. JAMA Dermatol.

[5] Paudel A, Dhital R, Areoye G, Basnet S, Tachamo N (2020). Sweet's syndrome in a granulocytopenic patient with acute myeloid leukemia on FLT3 inhibitor. J Community Hosp Intern Med Perspect.

[6] Cannas G, Pautas C, Raffoux E (2012). Infectious complications in adult acute myeloid leukemia: analysis of the Acute Leukemia French Association-9802 prospective multicenter clinical trial. Leuk Lymphoma.

[7] Lepelletier C, Bouaziz JD, Rybojad M, Bagot M, Georgin-Lavialle S, Vignon-Pennamen MD (2019). Neutrophilic dermatoses associated with myeloid malignancies. Am J Clin Dermatol.

[8] Wallach D, Vignon-Pennamen MD (2006). From acute febrile neutrophilic dermatosis to neutrophilic disease: forty years of clinical research. J Am Acad Dermatol.

[9] Marzano AV, Borghi A, Meroni PL, Cugno M (2016). Pyoderma gangrenosum and its syndromic forms: evidence for a link with autoinflammation. Br J Dermatol.

[10] Maalouf D, Battistella M, Bouaziz JD (2015). Neutrophilic dermatosis: disease mechanism and treatment. Curr Opin Hematol.

[11] Haga N, Iwata H, Yamaguchi Y (2016). Mucocutaneous pyoderma gangrenosum due to trisomy 8 neutrophilic infiltrates in a patient with myelodysplastic syndrome. Br J Dermatol.

[12] Magro CM, Crowson AN (2003). The spectrum of cutaneous lesions in rheumatoid arthritis: a clinical and pathological study of 43 patients. J Cutan Pathol.

[13] Yosipovitch G, Hodak E, Feinmesser M, David M (2000). Acute Crohn's colitis with lobular panniculitis--metastatic Crohn's?. J Eur Acad Dermatol Venereol.

[14] Sutra-Loubet C, Carlotti A, Guillemette J, Wallach D (2004). Neutrophilic panniculitis. J Am Acad Dermatol.

[15] Fraticelli P, Benfaremo D, Cardinali M, Gabrielli A (2019). Atypical neutrophilic panniculitis as presentation of BCR-ABL1-negative chronic myeloid leukaemia. BMJ Case Rep.

[16] Langan SM, Groves RW, Card TR, Gulliford MC (2012). Incidence, mortality, and disease associations of pyoderma gangrenosum in the United Kingdom: a retrospective cohort study. J Invest Dermatol.

[17] von den Driesch P (1997). Pyoderma gangrenosum: a report of 44 cases with follow-up. Br J Dermatol.

[18] Binus AM, Qureshi AA, Li VW, Winterfield LS (2011). Pyoderma gangrenosum: a retrospective review of patient characteristics, comorbidities and therapy in 103 patients. Br J Dermatol.

[19] Su WP, Davis MD, Weenig RH, Powell FC, Perry HO (2004). Pyoderma gangrenosum: clinicopathologic correlation and proposed diagnostic criteria. Int J Dermatol.

[20] Maverakis E, Ma C, Shinkai K (2018). Diagnostic criteria of ulcerative pyoderma gangrenosum: a Delphi consensus of international experts. JAMA Dermatol.

[21] Jockenhöfer F, Wollina U, Salva KA, Benson S, Dissemond J (2019). The PARACELSUS score: a novel diagnostic tool for pyoderma gangrenosum. Br J Dermatol.

[22] Haag C, Hansen T, Hajar T, Latour E, Keller J, Shinkai K, Ortega-Loayza AG (2021). Comparison of three diagnostic frameworks for pyoderma gangrenosum. J Invest Dermatol.

[23] Maronese CA, Pimentel MA, Li MM, Genovese G, Ortega-Loayza AG, Marzano AV (2022). Pyoderma gangrenosum: an updated literature review on established and emerging pharmacological treatments. Am J Clin Dermatol.

